# Safety and immunogenicity of a freeze-dried, Vero cell culture-derived, inactivated Japanese encephalitis vaccine (KD-287, ENCEVAC®) versus a mouse brain-derived inactivated Japanese encephalitis vaccine in children: a phase III, multicenter, double-blinded, randomized trial

**DOI:** 10.1186/s12879-014-0744-4

**Published:** 2015-01-08

**Authors:** Ki Wook Yun, Hoan Jong Lee, Jin Han Kang, Byung Wook Eun, Yae-Jean Kim, Kyung-Hyo Kim, Nam Hee Kim, Young Jin Hong, Dong Ho Kim, Hwang Min Kim, Sung-Ho Cha

**Affiliations:** Department of Pediatrics, Seoul National University College of Medicine, Seoul, Korea; Department of Pediatrics, Chung-Ang University College of Medicine, Seoul, Korea; Department of Pediatrics, College of Medicine, The Catholic University of Korea, Seoul, Korea; Department of Pediatrics, Eulji General Hospital, Eulji University School of Medicine, Seoul, Korea; Departments of Pediatrics, Samsung Medical Center, Sungkyunkwan University School of Medicine, Seoul, Korea; Department of Pediatrics, School of Medicine, Ewha Womans University, Seoul, Korea; Department of Pediatrics, Ilsan Paik Hospital, Inje University College of Medicine, Goyang, Korea; Department of Pediatrics, Inha University College of Medicine, Incheon, Korea; Department of Pediatrics, Korea Cancer Center Hospital, Seoul, Korea; Department of Pediatrics, Yonsei University Wonju College of Medicine, Wonju, Korea; Department of Pediatrics, College of Medicine, Kyunghee University, Seoul, Korea

**Keywords:** Japanese encephalitis, Vaccine, Vero cells, Clinical trial

## Abstract

**Background:**

Although mouse brain-derived, inactivated Japanese encephalitis vaccines (JE-MBs) have been successfully used for a long time, potential rare neurological complications have prompted the development of a Vero cell culture-derived inactivated vaccine (JE-VC). In a phase III clinical study, we aimed to compare the safety and immunogenicity of a JE-VC, KD-287 with a JE-MB, JEV-GCC, in children.

**Methods:**

In this multicenter, double-blinded, randomized controlled trial, the study population consisted of 205 healthy Korean children aged 12–23 months. Each subject was subcutaneously vaccinated with either KD-287 or JEV-GCC twice at an interval of 2 weeks and then vaccinated once 12 months after the second vaccination. Neutralizing antibodies were measured by the plaque reduction neutralization test using the homologous and heterologous, as a *post hoc analysis*, challenge virus strains.

**Results:**

The three-dose regimen of KD-287 showed a comparable safety profile with JEV-GCC except higher incidence of fever after the first dose (30.4% and 14.7%, respectively). Most of the fever was mild degree (61.3% and 66.7%, respectively). KD-287 fulfilled the non-inferiority criteria for seroconversion rate (SCR) and geometric mean titer (GMT) of the neutralizing antibody, which were the primary endpoints, at 4 weeks after the third vaccination (95% CI: −1.00, 3.10 for the SCR difference and 10.8, 17.6 for the GMT ratio). The SCRs of KD-287 were all 100% and the GMTs were higher in the KD-287 group than in the JEV-GCC group after the second vaccination and before and after the third vaccination (GMT ratio: 5.59, 20.13, and 13.79, respectively, *p* < 0.001 in all). GMTs were higher in the KD-287 group in the heterologous analysis also (GMT ratio: 4.05, 5.15, and 4.19, respectively, *p* < 0.001 in all).

**Conclusions:**

This study suggests that the KD-287, a JE-VC is as safe as and may be more effective than the licensed MB-derived vaccine. KD-287 could thus be useful as a second-generation vaccine and substitute for the current JE-MB vaccine in Korean children.

**Trial registration:**

ClinicalTrials.gov: NCT01150942

**Electronic supplementary material:**

The online version of this article (doi:10.1186/s12879-014-0744-4) contains supplementary material, which is available to authorized users.

## Background

Japanese encephalitis (JE) is the leading cause of severe viral infection of the central nervous system, mostly in Asian children. Japanese encephalitis viruses (JEVs) are transmitted by bird-biting mosquitoes, especially mosquitoes from the culex genus. There is no specific antiviral treatment for JE, and vaccination is the single most important control measure [1,2]. Mouse brain-derived, inactivated JE vaccines (JE-MBs) are available and are internationally accepted. Two strains of JEV, the Nakayama and the Beijing-1 strains, are used for the production of JE-MBs. Rare but serious hypersensitivity reactions and neurological complications have been reported following immunization with JE-MBs, potentially resulting from the presence of gelatin and murine neural proteins in the vaccines [3].

In 2009, an inactivated, Vero cell-derived JE vaccine (JE-VC; IXIARO®, Intercell Biomedical, Livingston, UK) employing SA 14-14-2 strain, was licensed in Europe, the United States, and Australia. This vaccine has a lower potential risk of serious neurological adverse reactions because it does not contain impurities from mouse-brain-derived proteins. Two other JE-VCs prepared using Beijing-1 strain (JEBIK®V, Biken, Kagawa, Japan and ENCEVAC®, Chemo-Sero-Therapeutic Research Institute [Kaketsuken], Kumamoto, Japan) have been used in Japan [4,5]; however, only JE-MBs (Green Cross vaccine, Green Cross, Yongin, Korea and Boryung vaccine, Boryung Pharmaceutical, Seoul, Korea) and a live-attenuated virus vaccine (CD.JEVAX®, Chengdu Institute of Biological Products, Sichuan, China) have been used to immunize children in South Korea [6,7]. There is a need for a JE-VC in South Korea to provide sufficient vaccine supply and overcome vaccine safety issues.

Although JE vaccines are routinely used in children of toddler age in endemic areas, previous studies that reported the safety and immunogenicity of JE-VC compared with JE-MB were mainly adult and early childhood studies [8–12]. Furthermore, in a previous study of ENCEVAC® in Japanese children [12], a JE-VC was compared with a JE-MB prepared using a homologous JEV strain (Beijing-1). In this phase III non-inferiority study, we aimed to compare the safety and immunogenicity of a JE-VC, KD-287 (ENCEVAC®, Beijing-1 strain) with those of a licensed JE-MB, JEV-GCC (Green Cross vaccine, Nakayama strain) in Korean children aged 12–23 months.

## Methods

### Participants

In this multicenter, double-blind, centrally randomized controlled trial, the study population consisted of healthy Korean children aged 12–23 months. The exclusion criteria consisted of impaired immunologic function; underlying chronic diseases; previous immunization for JEV; any investigational or non-registered drug use (during the study period), blood derivative use, immunosuppressant use, other immune-modifying drug use, or other vaccine administration within 4 weeks preceding the first dose of the study vaccine; history of encephalitis, meningitis, or other neurological disease; and a previous history of allergic reaction to components of the study vaccines. The study was performed in accordance with the principles outlined in the Declaration of Helsinki. Written informed consent was obtained from each parent or guardian after the possible consequences of the studies had been fully explained. The study protocol was approved by the Institutional Review Board of Seoul National University Hospital and the Korean Food and Drug Administration, as well as independent review board at each study site.

The intention-to-treat (ITT) population comprised all participants who were randomized to a study group, and the safety population comprised all participants receiving at least one vaccination. The per-protocol (PP) population for analysis of immunogenicity consisted of the participants who met all eligibility criteria, had completed all vaccinations, and had one pre-vaccination and three post-vaccination blood samples.

### Procedures

#### Vaccines

The JE test vaccine (KD-287) is a purified, freeze-dried, inactivated vaccine containing the JEV strain Beijing-1, which was adapted to grow in Vero cells. One vaccine dose contained 4 μg/0.5 mL of purified and inactivated virus, and it did not contain preservatives (such as thimerosal). KD-287 contained lactose, sodium phosphate, polysorbate, and glycine as stabilizers. Immediately prior to inoculation, a vial of KD-287 was reconstituted with 0.7 mL of distilled water.

The JE control vaccine (JEV-GCC) is a purified, liquid, inactivated vaccine containing the JEV strain Nakayama. This vaccine was derived from mouse brain tissue, and one dose contained 80 μg of protein/1 mL and thimerosal as a preservative.

#### Clinical study

The clinical study was conducted in accordance with the Rules of Immunization Practice, the Vaccination Guidelines in South Korea, and Good Clinical Practice guidelines. A centralized and computerized procedure was used to randomly assign participants. The vaccines were prepared and injected at the study sites by staff members who were not blinded to treatment assignments; the participants and all other investigators and staff members remained blinded to treatment assignments throughout the trial. The vaccine volumes for children under 36 months of age were half of the volumes for children who were at least 36 months old and were either 0.25 mL for children randomized to KD-287 or 0.5 mL for children randomized to JEV-GCC at enrollment. Each subject was subcutaneously vaccinated in the deltoid area twice at an interval of 2 weeks and then vaccinated once 12 months after the second vaccination as the primary immunization series, based on the recommended schedule for conventional JE vaccines in South Korea. This trial is registered as a clinical trial (clinicaltrials.gov: NCT01150942), protocol number KD287-BR-CT-301.

#### Safety

The children were observed for 30 min after inoculation of each dose by the investigators, and a parent/guardian was required to complete a diary card from days 0 to 7 to record daily signs and symptoms and daily temperature. Adverse events (AEs) were actively surveyed, and the occurrence of solicited local reactions (e.g., erythema, pain/tenderness, or swelling) and systemic symptoms (e.g., fever, crying, diarrhea, vomiting, somnolence, irritability, or decreased appetite) was documented. Onset date, end date, severity, action taken, outcome, and the investigator’s assessment of the relationship to the vaccination were recorded. All serious AEs (SAEs) between day 0 of the first vaccination and 6 months after the third vaccination were reported actively by parents/guardians or passively at each visit after the first vaccination during study period and the telephone interviews which were done at day 7 after each vaccination and 6 months after the third vaccination.

Investigators and parents/guardians used a standard scale to grade solicited AEs during the observation period. Fever was defined as mild if the body temperature was 37.5–38.4°C, moderate if 38.5–39.4°C, and severe if ≥39.5°C. Other systemic symptoms were considered mild if they did not interfere with normal daily activities, moderate if they resulted in some interference with normal daily activities, and severe if they prevented subjects from engaging in normal daily activities. Erythema and swelling at the injection site were defined as mild if the diameter was <10 mm, moderate if 10–29 mm, and severe if ≥30 mm.

#### Immunogenicity

For measurement of neutralizing antibody titers, serum was obtained from each subject before the first and third vaccination and 4 weeks after the second and third vaccination; the serum was stored at −80°C until the time of measurement. Levels of neutralizing antibodies were measured by the plaque reduction neutralization test (PRNT), the standard method for evaluating immunogenicity in JE vaccine trials [13,14]. The neutralizing antibody titer was expressed as a reciprocal of the dilution of serum that produced a 50% reduction of plaque formation (PRNT_50_) relative to the plaque number of the diluted challenge virus in the absence of antiserum.

As the study vaccines contained different JEV strains (Beijing-1 in KD-287 and Nakayama in JEV-GCC), serum samples of the two groups were individually tested with the corresponding vaccine strain as a challenge virus in each sample (homologous analysis). In addition, as a *post hoc* analysis, we also tested all serum samples with the opposite vaccine strain as a challenge virus (heterologous analysis) to avoid potential bias in favor of either strain [15].

Seropositivity was defined as a PRNT_50_ titer of 1:10 or greater; this cut-off was established in animal experiments [13]. The serum samples were tested at a starting dilution of 1:10, then 4-fold serial dilutions up to 1,280, or higher when needed, and the negative samples were assigned a value of 1:5 for calculation purposes. A correlation of neutralizing antibody titer with protective efficacy was shown in a phase III study in Thailand supporting the licensing of an inactivated JE-MB (JE-VAX) in the United States [16]. Seroconversion was defined as a change from seronegative to seropositive or a 4-fold increase in the neutralizing antibody titer for a seropositive subject before vaccination.

#### Statistical analysis

The primary objective of the study was to show the non-inferiority of the test vaccine compared with the licensed control vaccine in terms of primary immunogenicity endpoints, as the homologous response. The secondary objective was to evaluate and compare the safety and tolerability of the two vaccines. Primary endpoints were seroconversion rate (SCR) and geometric mean titer (GMT) at 4 weeks after the third vaccination, and the secondary endpoints were the SCRs and GMTs at 4 weeks after the second vaccine dose and before the third vaccine dose. Non-inferiority of the test vaccine would be shown if the lower limit of the 95% confidence interval (CI) for the seroconversion difference (i.e., test vaccine minus licensed vaccine) was higher than −10% and if the lower limit of the 95% CI for the GMT ratio (i.e., test vaccine divided by licensed vaccine) was higher than 0.5. Their 95% CIs were computed by transforming the results to a logarithmic scale [14]. All immunogenicity data in the heterologous response were additionally obtained in the *post hoc* analysis.

We calculated that 200 participants would be needed to show non-inferiority of the SCR with a presumptive drop-out rate of 25%, a two-sided 95% CI, and a power of 80%, assuming a SCR of 95% in both groups. All immunogenicity assessments were performed on both the PP (n = 188) and ITT (n = 205) populations. Because there are no significant differences between the two populations, the data of PP population are shown. The safety of the vaccines was assessable in the children in the safety population (n = 204). The incidence rates of AE were estimated as the case number (n) and proportion (%) and were compared using the chi-square or Fisher’s exact test to determine whether there was a difference between the treatment groups. All statistical analyses were performed using SAS version 9.2 (SAS Institute Inc., Cary, NC, USA).

## Results

### Study population

**Figure 1:**
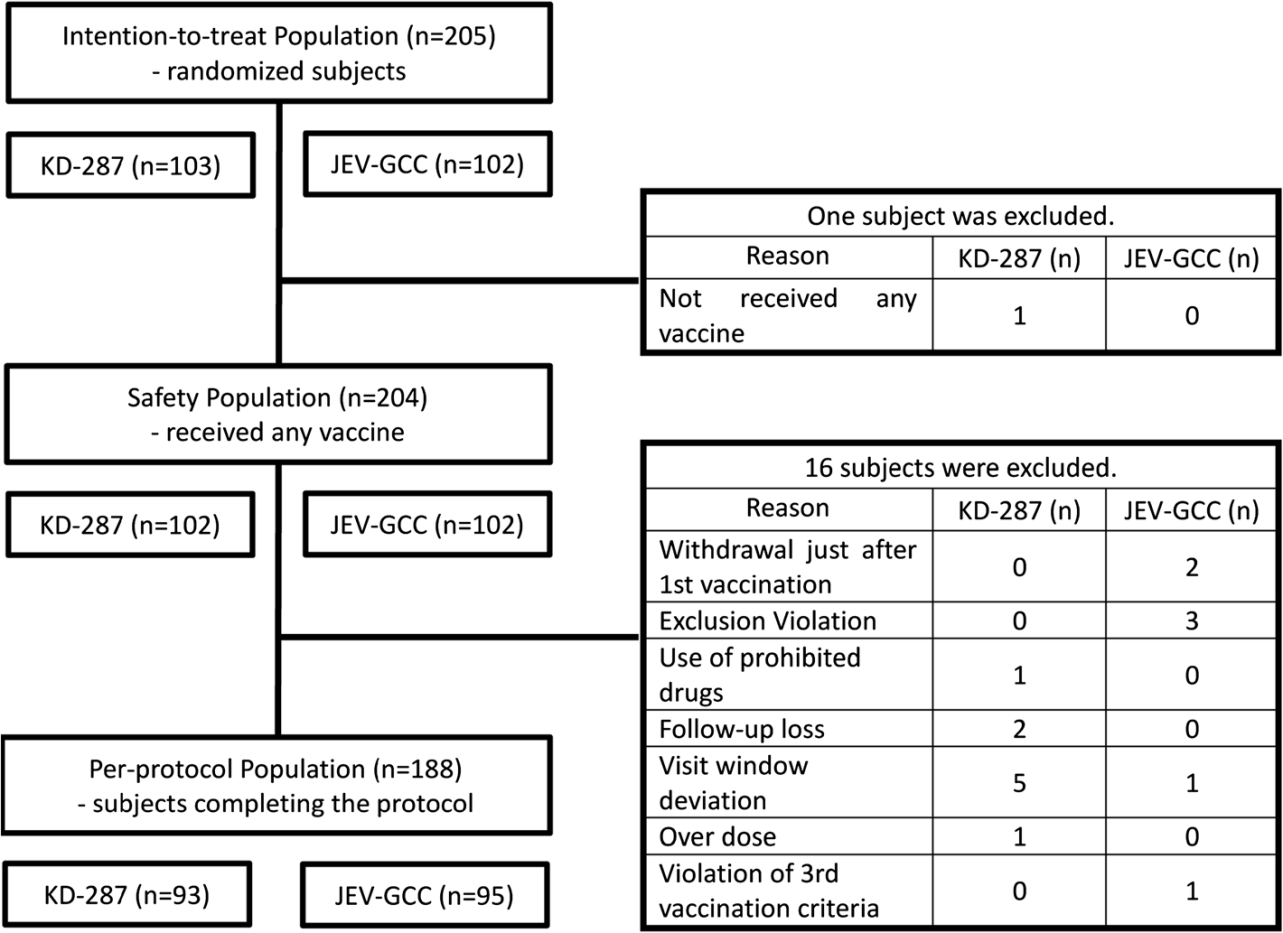
**Analysis populations and excluded subjects.**

This study enrolled 205 participants across 10 centers: 103 participants were randomized to KD-287 and 102 to JEV-GCC, 188 (91.7%; PP population) of whom (93/103 [90.3%] in the test group and 95/102 [93.1%] in the control group) completed the study up to 6 months after the third vaccination (Figure 1). Seventeen (8.3%) children were dropped because of eligibility criteria violations (n = 3; other vaccination 2 weeks before enrollment [n = 1] and underlying chronic diseases [n = 2]), follow-up loss (n = 2), withdrawal of consent (n = 3), and protocol violation before the third vaccination (n = 9; prohibited drug use [n = 1], visit window deviation [n = 6], over dose [n = 1], and violation of third vaccination criteria [neurologic SAE; n = 1]). All participants who decided not to continue with the study withdrew their consent for personal reasons. All of the randomized participants (n = 205, 100%; ITT population) except one, who voluntarily withdrew before the first vaccination, were included in the safety population (n = 204, 99.5%).

### Adverse events after vaccination

The overall rates of solicited AEs for KD-287 and JEV-GCC, which are the proportions of subject having at least one AE after any vaccination, were 85.3% (87/102) and 75.5% (77/102), respectively (*p* = 0.050, Table 1). Most AEs were mild to moderate in both groups (75/87 [86.2%], KD-287; 67/77 [87.0%], JEV-GCC). The solicited AE rates associated with the first, second, and third injections of KD-287 were 63.7% (65/102), 44.1% (45/102), and 46.0% (46/100), respectively, whereas the corresponding rates for JEV-GCC were 52.9% (54/102), 45.0% (45/100), and 52.1% (50/96), respectively; there were no significant differences between the two groups when compared at each vaccination (*p* = 0.118, 0.900, and 0.394, respectively).

**Table 1:** **Solicited adverse events after vaccinations at each dose in the study subjects (safety population)**

	**Group (n)**	**No. (%) of subjects**		***p*** -value*
		**AE**	**no AE**	
Total	KD-287 (102)	87 (85.29)	15 (14.71)	0.050
	JEV-GCC (102)	77 (75.49)	25 (24.51)	
After 1^st^ Vaccination	KD-287 (102)	65 (63.73)	37 (36.27)	0.118
	JEV-GCC (102)	54 (52.94)	48 (47.06)	
After 2^nd^ Vaccination	KD-287 (102)	45 (44.12)	57 (55.88)	0.900
	JEV-GCC (100)	45 (45.00)	55 (55.00)	
After 3^rd^ Vaccination	KD-287 (100)	46 (46.00)	54 (54.00)	0.394
	JEV-GCC (96)	50 (52.08)	46 (47.92)	

AE, adverse event.

*The *p*-value was calculated using the chi-square test.

All solicited AEs occurring within 7 days after each vaccination in both groups are shown in Table 2. Overall, the local tolerability and systemic safety profile of KD-287 was promising in the study, with no serious safety concerns identified. The most common AE indicated by participants was crying in mild severity (27/102 [26.5%], KD-287; 20/102 [19.6%], JEV-GCC) after first vaccination. The frequency of most AEs was similar between the two groups and most local and systemic events were mild or moderate in severity. Only the total incidence of fever after the first vaccination was significantly different between the two groups (31/102 [30.4%], KD-287; 15/102 [14.7%], JEV-GCC, *p* = 0.012). Among the fever events after the first vaccination in KD-287 and JEV-GCC groups, 61.3% (19/31) and 66.7% (10/15) were mild severity, respectively.

**Table 2:** **Incidence of solicited adverse events within 7 days after each vaccination (safety population)**

	**Severity**	**1** ^**st**^ dose, n*(%)		**2** ^**nd**^ dose, n*(%)		**3** ^**rd**^ dose, n*(%)	
		**KD-287 (n = 102)**	**JEV-GCC (n = 102)**	**KD-287 (n = 102)**	**JEV-GCC (n = 100)**	**KD-287 (n = 100)**	**JEV-GCC (n = 96)**
Erythema	Mild	9 (8.8)	18 (17.6)	7 (6.9)	14 (14.0)	7 (7.0)	14 (14.6)
	Moderate	3 (2.9)	2 (2.0)	5 (4.9)	2 (2.0)	7 (7.0)	6 (6.3)
	Severe	1 (1.0)	0 (0)	0 (0)	0 (0)	1 (1.0)	0 (0)
Pain	Mild	14 (13.7)	10 (9.8)	3 (2.9)	5 (5.0)	18 (18.0)	11 (11.5)
	Moderate	2 (2.0)	2 (2.0)	2 (2.0)	1 (1.0)	2 (2.0)	3 (3.1)
	Severe	0 (0)	0 (0)	0 (0)	0 (0)	0 (0)	1 (1.0)
Swelling	Mild	3 (2.9)	7 (6.9)	6 (5.9)	7 (7.0)	5 (5.0)	11 (11.5)
	Moderate	2 (2.0)	1 (1.0)	2 (2.0)	1 (1.0)	1 (1.0)	2 (2.1)
	Severe	1 (1.0)	0 (0)	0 (0)	0 (0)	1 (1.0)	0 (0)
Fever	Mild	19 (18.6)	10 (9.8)	13 (12.7)	12 (12.0)	13 (13.0)	14 (14.6)
	Moderate	8 (7.8)	4 (3.9)	2 (2.0)	4 (4.0)	1 (1.0)	3 (3.1)
	Severe	4 (3.9)	1 (1.0)	2 (2.0)	3 (3.0)	0 (0)	1 (1.0)
Crying	Mild	27 (26.5)	20 (19.6)	15 (14.7)	6 (6.0)	14 (14.0)	14 (14.6)
	Moderate	5 (4.9)	4 (3.9)	3 (2.9)	6 (6.0)	2 (2.0)	3 (3.1)
	Severe	2 (2.0)	0 (0)	0 (0)	1 (1.0)	0 (0)	1 (1.0)
Irritability	Mild	12 (11.8)	11 (10.8)	5 (4.9)	6 (6.0)	13 (13.0)	4 (4.2)
	Moderate	1 (1.0)	4 (3.9)	2 (2.0)	5 (5.0)	0 (0)	6 (6.3)
	Severe	0 (0)	0 (0)	0 (0)	0 (0)	0 (0)	0 (0)
Vomiting	Mild	8 (7.8)	11 (10.8)	4 (3.9)	3 (3.0)	1 (1.0)	4 (4.2)
	Moderate	2 (2.0)	0 (0)	0 (0)	0 (0)	0 (0)	0 (0)
	Severe	1 (1.0)	0 (0)	0 (0)	0 (0)	0 (0)	0 (0)
Diarrhea	Mild	17 (16.7)	11 (10.8)	3 (2.9)	5 (5.0)	3 (3.0)	4 (4.2)
	Moderate	4 (3.9)	1 (1.0)	0 (0)	2 (2.0)	0 (0)	2 (2.1)
	Severe	0 (0)	1 (1.0)	0 (0)	0 (0)	1 (1.0)	1 (1.0)
Decreased appetite	Mild	10 (9.8)	13 (12.7)	5 (4.9)	8 (8.0)	6 (6.0)	8 (8.3)
	Moderate	2 (2.0)	0 (0)	0 (0)	1 (1.0)	1 (1.0)	0 (0)
	Severe	2 (2.0)	0 (0)	1 (1.0)	0 (0)	0 (0)	1 (1.0)
Somnolence	Mild	9 (8.8)	12 (11.8)	8 (7.8)	7 (7.0)	14 (14.0)	17 (17.7)
	Moderate	0 (0)	1 (1.0)	0 (0)	1 (1.0)	0 (0)	0 (0)
	Severe	0 (0)	0 (0)	0 (0)	0 (0)	0 (0)	0 (0)

*Number of subjects reporting the specific characteristic.

In total, 52 SAEs were reported: 23 occurred in 17 children in the KD-287 group and 29 occurred in 23 children in the JEV-GCC group (*p* = 0.373, Table 3). These events were regarded as most likely not related to the vaccine itself except for one event of febrile seizure in the KD-287 group. No vaccine-related hypersensitivity or neurological AE was reported.

**Table 3:** **Incidence of serious adverse events requiring hospitalization (safety population)**

**Adverse events**	**No. of subjects, n (%)**		***p*** -value*
	**KD-287 (n = 102)**	**JEV-GCC (n = 102)**	
Total	17 (16.67)	23 (22.55)	0.373
Febrile seizure	1 (0.98)	-	1.000
Otitis Media	1 (0.98)	1 (0.98)	1.000
Pharyngitis	2 (1.96)	1 (0.98)	1.000
Laryngitis	1 (0.98)	-	1.000
Bronchitis	4 (3.92)	4 (3.92)	1.000
Pneumonia	5 (4.90)	13 (12.75)	0.127
Influenza	-	1 (0.98)	1.000
Gastroenteritis	1 (0.98)	3 (2.94)	0.369
Others^†^	2 (1.96)	-	0.498

*The *p*-value was calculated using the chi-square or Fisher’s exact test.

^†^Others included a case of vaccine overdose and a case of accessory skin tag.

### Immunogenicity

The immunogenicity population comprised 188 participants: 93 in the KD-287 group and 95 in the JEV-GCC group. The SCRs and GMTs for each group after vaccination in the homologous analysis are shown in Table 4. Before vaccination, none of the children, except for one in the JEV-GCC group, had detectable antibodies against JEV.

**Table 4:** **Seroconversion rates and geometric mean titers after vaccination compared to before vaccination (per-protocol population, homologous response)**

	**Time point**	**Value**	**KD-287 (n = 93)**	**JEV-GCC (n = 95)**	*Difference/Ratio
SCR	Seropositive at baseline	n (%)	0 (0.0)	1 (1.0)	
		95% CI			
	After 2^nd^ dose	n (%)	93 (100.0)	93 (97.9)	2.1
		95% CI			(−0.78, 4.99)
	Before 3^rd^ dose	n (%)	93 (100.0)	90 (94.7)	5.3
		95% CI			(0.77, 9.75)
	After 3^rd^ dose	n (%)	93 (100.0)	94 (98.9)	1.1
		95% CI	(100.0, 100.0)	(96.9, 100.0)	(−1.00, 3.10)
GMT	Before Vaccination	log10^n^	5	5	1.0
		95% CI	(5, 6)		(1.0, 1.1)
	After 2^nd^ dose	log10^n^	601	107	5.6^†^
		95% CI	(509, 709)	(84, 137)	(4.2, 7.5)
	Before 3^rd^ dose	log10^n^	917	45	20.1^†^
		95% CI	(757, 1110)	(36, 56)	(15.2, 26.7)
	After 3^rd^ dose	log10^n^	13347	967	13.8^†^
		95% CI	(11455, 15551)	(798, 1172)	(10.8, 17.6)

SCR, seroconversion rate; GMT, geometric mean titer; CI, confidence interval.

*Difference is KD-287 minus JEV-GCC for SCR and ratio is KD-287 divided by JEV-GCC for GMT.

^†^The *p*-value was calculated using the *t*-test for GMT; *p* < 0.001 in all.

The immunogenicity assessed by SCRs and GMTs at 4 weeks after the third injection was excellent in both vaccines. Only one subject in JEV-GCC group was not seroconverted to JEV. The GMTs were higher in the KD-287 group than they were in the JEV-GCC group after the third vaccination (GMT ratio: 13.79, *p* < 0.001). Because the lower limit of the 95% CI for the seroconversion difference (−1.00%) and for the GMT ratio (10.8) was higher than −10% and 0.5, respectively, we concluded that KD-287 achieved the non-inferiority criteria.

Furthermore, the SCRs after the second vaccination and before the third vaccination were excellent in both groups. The GMTs were higher in the KD-287 group than they were in the JEV-GCC group after the second vaccination and before the third vaccination (GMT ratio: 5.59 and 20.13, respectively, *p* < 0.001 in both). The reverse cumulative curve of neutralizing antibodies also revealed that immune responses of the KD-287 group were greater compared to those of the JEV-GCC group after the second vaccination and before and after the third vaccination, respectively (see Additional file 1: Figure S1).

In the heterologous analysis, the SCRs after the third injection were excellent in both vaccines, so that only one subject in KD-287 group was not seroconverted (see Additional file 2: Table S1). The SCRs in the KD-287 group were higher than those in the JEV-GCC group, both after the second vaccination (94.62% and 54.74%, respectively) and before the third vaccination (93.55% and 34.74%, respectively). Against the heterologous strain, the GMTs were higher in the KD-287 group than they were in the JEV-GCC group after the second vaccination and before and after the third vaccination (GMT ratio: 4.05, 5.15, and 4.19, respectively, *p* < 0.001 in all). The lower limits of the 95% CI both for the seroconversion difference (−3.2) and for the GMT ratio (3.2) in the heterologous analysis also fulfilled the non-inferiority criteria.

**Figure 2:**
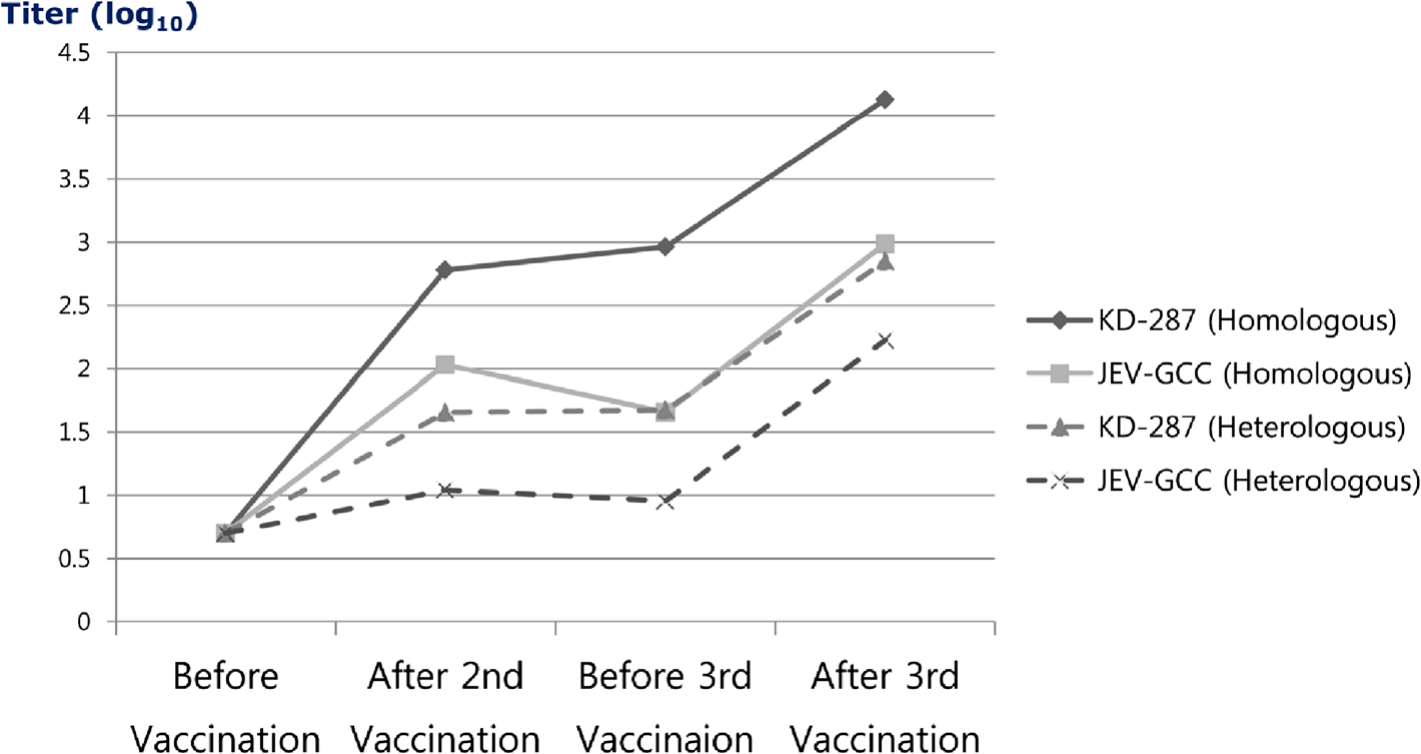
**Changes of mean neutralizing antibody titers before and after vaccination.** Satisfactory geometric mean titers (GMTs) were obtained after the second vaccination and before and after the third vaccinations in both vaccine groups, in both homologous and heterologous analyses. The GMTs before the third vaccination in the KD-287 group were higher than those after the second vaccination for both tested strains. After the third vaccination, the neutralizing antibody titer increased in all children, and the GMTs in both groups were higher than the respective GMTs after the second vaccination.

Before the third vaccination, children in the JEV-GCC group showed slightly reduced GMTs compared with the GMTs after the second vaccination, but satisfactory GMTs were obtained against both homologous and heterologous strains (Figure 2). However, the GMTs before the third vaccination in the KD-287 group were higher than the GMTs after the second vaccination for both tested strains. After the third vaccination, the neutralizing antibody titer increased in all children, and the GMTs in both groups were higher than the respective GMTs after the second vaccination.

## Discussion

We performed a phase III clinical study of a JE-VC prepared with the Beijing-1 strain, KD-287, with the aim of determining whether its immunogenicity is not inferior and its safety is comparable to those of the currently used JE-MB prepared with the Nakayama strain, JEV-GCC, in Korean children. Statistical non-inferiority of KD-287 to JEV-GCC was demonstrated in terms of SCRs and GMTs at 4 weeks after the third vaccination. Neutralizing antibody titers were significantly higher in the KD-287 group than in the JEV-GCC group after the second vaccination and before and after the third vaccination. The safety profile of KD-287 was good, and its local and systemic tolerability profiles were comparable to those of JEV-GCC, except for a higher incidence of fever after the first vaccination. The frequency of AEs was similar between the two groups, and vaccine-related AEs were generally mild. There was no notable SAE, including neurologic symptoms, in the two vaccine groups.

JEV is regarded as one of the most serious viral causes of encephalitis, with a mortality of 30% and a high rate of neurologic sequelae in survivors [1]. Because no antiviral treatment is available, prevention of infection is particularly important. Routine immunization programs against JE are generally recommended for populations residing in endemic areas. In developed, non-endemic countries, vaccinations are recommended for travelers to endemic regions [2,16].

The Nakayama strain of the JEV was isolated from the cerebral spinal fluid of a patient in 1935 and was maintained by continuous mouse-brain passage. The Nakayama strain JE-MB has been the dominant vaccine throughout Asia, with the exception of the Beijing strain product, which was produced in Japan from 1988 to 2005 [16,17]. The residual mouse brain neural tissue content of the vaccine has raised concerns regarding safety and the possibility of vaccine-related neurological adverse events [18,19]. Furthermore, the manufacture of the vaccine is associated with difficulties in obtaining a large supply of mice, high cost, and ethical issues related to the use of animals. At present, JE-VCs, live-attenuated virus vaccines, and recombinant chimeric virus vaccines (JE-CVs) are available as second-generation JE vaccine alternatives to JE-MB [4].

Sugawara et al. found that Vero cells show a high susceptibility to JEV and that the virus shows good proliferation in cultured cells [20]. They also confirmed that the viral antigens derived from mouse brain and Vero cells have similar physicochemical and immunological characteristics and that Vero cell-derived antigens could be used as a vaccine. The IC51 vaccine (IXIARO®) is a formalin-inactivated JE-VC prepared with the whole-virus SA 14-14-2 strain that was developed by Intercell Biomedical (Livingston, UK) [20]. IC51 has been licensed and indicated for prevention of JE in individuals aged ≥2 months in Europe and the United States. Recently, a JE-VC similar to IC51 became available in India and is manufactured by Biological E using technology transferred from Intercell [21,22]. In Japan, freeze-dried, inactivated JE-VCs prepared with the Beijing-1 strain are distributed by two manufacturers: Biken (JEBIK V®; BK-VJE) and the Chemo-Sero-Therapeutic Research Institute (ENCEVAC®; KD-287). These two JE-VCs were approved in Japan as a substitute for JE-MB in 2009 and 2011, respectively, and they are indicated for prevention of JE in individuals aged ≥6 months [4,10,23]. However, in South Korea, only JE-MBs locally produced using the Nakayama strain and a live-attenuated virus vaccine prepared with the SA 14-14-2 strain have been used for immunization of children [6,7].

Although several JE vaccines are in use today, only the formalin-inactivated JE vaccine has been evaluated in placebo-controlled trials [16,24]. After the efficacy of purified JE-MB vaccines was demonstrated in these studies, placebo-controlled JE vaccine efficacy trial was considered unethical. In addition, a comparative trial between a licensed vaccine and a new vaccine using the endpoint of prevention of clinical illness would require an unfeasibly large sample size. Hence, there is a need to determine a vaccine-induced immunological marker that may be used as a surrogate to indicate protection from disease [14]. It has been established that neutralizing antibodies provide the best evidence of protective immunity, and functional assays of neutralization show a correlation with protection. A linear titer-protection relationship exists, and data from efficacy trials corroborate the role of neutralizing antibodies in protection [14,25].

The immunogenicity and safety of a 2-dose primary series of IC51 (IXIARO®) has been demonstrated for adults in two large company-coordinated, non-inferiority randomized controlled trials (RCTs) [8,9]. In addition to having a favorable safety and tolerability profile, the SCR induced by two IC51 (SA 14-14-2 strain) doses (98%) was demonstrated to be non-inferior to the SCR induced by three doses of a JE-MB (Nakayama strain) (95%). Furthermore, the GMT after IC51 vaccination was 2-fold higher than that after JE-MB vaccination, although GMTs were evaluated against the homologous strain for IC51 and the heterologous strain for JE-MB [8]. The immunogenicity and safety of IC51 were then evaluated in healthy children between 1 and 3 years of age in a phase II study [11]. At 56 days after vaccination, the SCR of the 3 μg and 6 μg IC51 groups and the JE-MB (Nakayama strain) group were 95.7%, 95.2%, and 90.9%, respectively (*p* > 0.05). There was no apparent difference in the safety profile between the vaccines. Recently, it was shown that a single dose of IC51 effectively boosted immunity in adults primed with a JE-VC [26,27] or a JE-MB [15,28].

Another JE-VC, BK-VJE (Beijing-1 strain) was recently evaluated for immunogenicity and safety in Japanese children aged 6–90 months [10,23]. BK-VJE at one half of the potency of a JE-MB (Beijing-1 strain) was sufficient to induce the comparable neutralizing antibody titer to JE-MB at full potency, and there was no withdrawal of subjects as a result of vaccine-related adverse events.

In addition, two RCTs with KD-287 were conducted in healthy children aged 6–90 months in Japan [12]. The KD-287 was in liquid form in one study and in freeze-dried form in the other study. Both forms of the test vaccine and control vaccine (liquid form, JE-MB prepared with the Beijing-1 strain) were injected twice at a 1–4 week interval, and the third dose was injected 1–15 months later. After the third injection, the SCRs in the KD-287 and the control vaccine groups were 100% in both studies. The GMTs after the second and third injection were both significantly higher in the KD-287 group than those in the control vaccine group in both studies. Although there were no important safety issues in both studies, low-grade fever was more prevalent in the KD-287 group than in the control vaccine group. In this study, we performed a double-blind phase III RCT to assess the safety and immunogenicity of KD-287 in Korean healthy children aged 12–23 months. The two vaccines were different with regard to the method used for JEV propagation (Vero cells vs. mouse brain) and were also different with regard to the JEV strain included (Beijing-1 vs. Nakayama).

All JEV strains belong to a single serotype and are classified in five genotypes. All currently available JE vaccines are prepared from genotype III strains, and the JEV strains prevalent in South Korea are mostly from genotype I, and more recently genotype V [29]. However, the genotype of JEV strain in a JE vaccine may not affect the protective efficacy of the vaccine [25]. In most previous JE vaccine immunogenicity studies, only the homologous virus strain was used in PRNT assays for each vaccine [8,11,12,23,26–28]. In the studies where the virus strains of the test and control vaccines were heterologous, the PRNT assay favored the vaccine homologous to the target strain [15,30–32]. The JE vaccine prepared with the Beijing-1 strain induced higher levels of neutralizing antibodies against heterologous JE strains than the JE vaccine prepared with the Nakayama strain. However, JE vaccines prepared using either the Beijing-1 or Nakayama strains can induce effective protective immunity against heterologous JEV strains. We tested all serum samples against both of the vaccine strains included in JE-MB and JE-VC (Nakayama and Beijing-1) as previously suggested [15].

JE-VC and JE-MB are thought to have similar properties; however, some differences exist in the vaccine manufacturing processes. For instance, the inactivation and purification conditions for JE-VC are milder than those used for JE-MB. Electron microscopy showed that JE-MB contains virions with a somewhat smooth surface, whereas the surface of JE-VC virus particles is more similar to that of the native virus. Thus, the conformation of the E protein on the virus surface may be better conserved in JE-VC, which may be important for the superior immunogenicity of JE-VC [10].

In this study, the SCRs after a three-dose injection of the KD-287 were excellent in both homologous and heterologous analyses. Compared with the licensed control vaccine, KD-287 fulfilled the non-inferiority criteria. Furthermore, the GMTs at three test points after vaccination of KD-287 were significantly higher than those of the control vaccine in both homologous and heterologous analyses. These results agree with the findings of previous RCTs comparing JE-VC with JE-MB [8,10,12,23]. Non-inferiority of KD-287 to the control vaccine with regard to the GMT ratio was also confirmed in this study. Interestingly, the GMTs before the third vaccination were higher than those after the second vaccination in the KD-287 group. Because there was an 11-month interval between the second and third samplings, the GMTs were generally expected to decrease temporally. It is possible that we did not select the time point with peak neutralizing antibody production after the second JE vaccination, especially in the KD-287 group. Neutralizing antibody production may continue beyond 4 weeks after the second injection; thus, the peak GMTs after the second vaccination may actually be higher than the values we observed in the KD-287 group. Previous studies showed approximately two-fold higher GMTs for JE-VC than for JE-MB [8,10,23], but the GMTs after three doses of JE-VC prepared with Beijing-1 strain JEV were approximately 14-fold higher than those of JE-MB prepared with Nakayama strain JEV in this study. We speculate that the Beijing-1 strain of JEV may be more immunogenic than the Nakayama strain of JEV in healthy Korean children.

The safety and tolerability with the KD-287 were excellent in this study. As shown in Table 1, the rate of solicited AEs reported after each dose was not significantly different between the two vaccines, although the total incidence of solicited AEs in the KD-287 group was higher than that in the control vaccine group (borderline statistical significance [*p* = 0.050]). Furthermore, severe AEs were infrequent in both vaccine groups (12/102 [13.8%] in the KD-287 group, 10/102 [13.0%] in the JEV-GCC group, *p* > 0.05). The rates of all solicited AEs within 7 days after each vaccination were also not significantly different between the vaccine groups, except the higher incidence of mild fever events after the first injection of KD-287 than that of JEV-GCC. As an increased incidence of mild fever for freeze-dried, but not liquid, KD-287 was also identified in a previous study [12], further efforts to identify and eliminate the cause of fever are necessary for KD-287.

The rates of all SAEs also did not differ between the vaccine groups. Only one febrile seizure event in the KD-287 group may have been associated with vaccination. Although there was remarkably high incidence of SAEs in both groups, it was possibly due to the high medical attention of parents of study subjects and specific situation in Korean healthcare system convenient to be hospitalized. There were no serious neurologic adverse reactions in the study and almost all children completed the three-dose regimen. The three-dose schedule of KD-287 will most likely have an acceptable tolerability with a low frequency of vaccine-related adverse reactions in children aged 12–23 months.

This study is limited by differences between the two vaccines in the method of JEV propagation (Vero cells vs. mouse brain) and in the JEV strains included (Beijing-1 vs. Nakayama). We could not measure the actual effect of the vaccines against JE in the children.

## Conclusions

In this study, we confirmed that KD-287, the JE-VC prepared with the Beijing-1 strain, showed a comparable safety profile with and a higher immunogenicity than the JE-MB prepared with the Nakayama strain. Furthermore, a positive booster effect was also recognized after a third dose of either vaccine. In conclusion, KD-287 could be useful as a second-generation vaccine and substitute for the currently used JE-MB vaccine in South Korea.

## Additional files

Additional file 1: Figure S1. Reverse cumulative curve of geometric mean titer in the study subjects (per-protocol population). Neutralizing antibody levels were evaluated using the Beijing-1 strain virus in the KD-287 group and the Nakayama strain virus in the JEV-GCC group (homologous analysis). Lines drawn with a character “x”, open circle (o), and rhombus (♦) indicate the antibody titers after the second vaccination, and before and after the third vaccination, respectively, in both groups. Lines for the JEV-GCC and KD-287 groups are drawn in blue and red, respectively. The levels of neutralizing antibodies against JEV were higher in the KD-287 group than in the JEV-GCC group after the second vaccination and before and after the third vaccination. Additional file 2: Table S1. Seroconversion rates and geometric mean titers after vaccination compared to before vaccination (per-protocol population, heterologous response).
