# Supplementary figures and images for: Safety and immunogenicity of a freeze-dried, Vero cell culture-derived, inactivated Japanese encephalitis vaccine (KD-287, ENCEVAC®) versus a mouse brain-derived inactivated Japanese encephalitis vaccine in children: a phase III, multicenter, double-blinded, randomized trial
Source: BMC Infect Dis. 2015 Jan 8;15:7. doi: 10.1186/s12879-014-0744-4 (PMC4296691; doi:10.1186/s12879-014-0744-4)

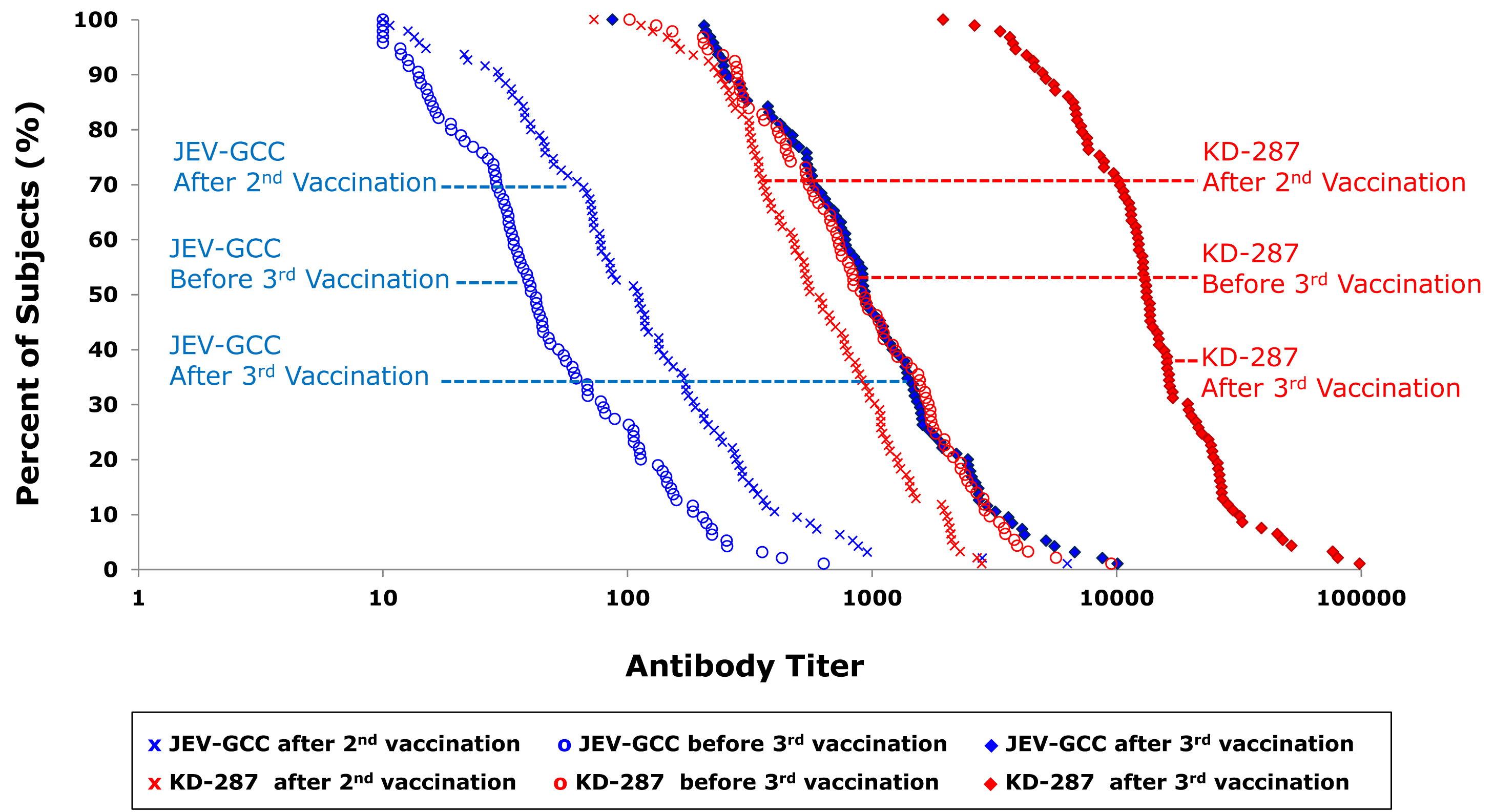

Supplement: Additional file 1: Figure S1. — Reverse cumulative curve of geometric mean titer in the study subjects (per-protocol population). Neutralizing antibody levels were evaluated using the Beijing-1 strain virus in the KD-287 group and the Nakayama strain virus in the JEV-GCC group (homologous analysis). Lines drawn with a character “x”, open circle (o), and rhombus (♦) indicate the antibody titers after the second vaccination, and before and after the third vaccination, respectively, in both groups. Lines for the JEV-GCC and KD-287 groups are drawn in blue and red, respectively. The levels of neutralizing antibodies against JEV were higher in the KD-287 group than in the JEV-GCC group after the second vaccination and before and after the third vaccination. [file 12879_2014_744_MOESM1_ESM.tiff]
