# Supplementary material for: Safety and immunogenicity of a freeze-dried, Vero cell culture-derived, inactivated Japanese encephalitis vaccine (KD-287, ENCEVAC®) versus a mouse brain-derived inactivated Japanese encephalitis vaccine in children: a phase III, multicenter, double-blinded, randomized trial
Source: BMC Infect Dis. 2015 Jan 8;15:7. doi: 10.1186/s12879-014-0744-4 (PMC4296691; doi:10.1186/s12879-014-0744-4)
Supplement: Additional file 2: Table S1. — Seroconversion rates and geometric mean titers after vaccination compared to before vaccination (per-protocol population, heterologous response). [file 12879_2014_744_MOESM2_ESM.doc]

Table S1 Seroconversion rates and geometric mean titers after vaccination compared to before vaccination (per-protocol population, heterologous response)

|  | Time point | Value | KD-287 (n=93) | JEV-GCC (n=95) | *Difference/Ratio |
| --- | --- | --- | --- | --- | --- |
| SCR | Seropositive  at baseline | n (%) | 0 (0.0) | 1 (1.0) |  |
|  |  | 95% CI |  |  |  |
|  | After 2^nd^ dose | n (%) | 88 (94.6) | 52 (54.7) | 39.9 |
|  |  | 95% CI |  |  | (28.88, 50.90) |
|  | Before 3^rd^ dose | n (%) | 87 (93.6) | 33 (34.7) | 58.8 |
|  |  | 95% CI |  |  | (48.01, 69.61) |
|  | After 3^rd^ dose | n (%) | 92 (98.9) | 95 (100.0) | -1.08 |
|  |  | 95% CI | (96.8, 100.0) | (100.0, 100.0) | (-3.17, 1. 20) |
| GMT | Before Vaccination | log10^n^ | 5 | 5 | 1.0 |
|  |  | 95% CI | (5, 6) |  | (1.0, 1.1) |
|  | After 2^nd^ dose | log10^n^ | 45 | 11 | 4.1**†** |
|  |  | 95% CI | (37, 53) | (9, 13) | (3.2, 5.2) |
|  | Before 3^rd^ dose | log10^n^ | 47 | 9 | 5.2**†** |
|  |  | 95% CI | (39, 57) | (8, 11) | (4.0, 6.7) |
|  | After 3^rd^ dose | log10^n^ | 705 | 168 | 4.2**†** |
|  |  | 95% CI | (591, 843) | (137, 207) | (3.2, 5.5) |

SCR, seroconversion rate; GMT, geometric mean titer; CI, confidence interval

*Difference is KD-287 minus JEV-GCC for SCR and ratio is KD-287 divided by JEV-GCC for GMT.

**†**The *p*-value was calculated using the *t*-test for GMT; *p*<0.001 in all.
